# On the Ground in Malawi—First Typhoid Conjugate Vaccine Study in Africa

**DOI:** 10.4269/ajtmh.19-0014

**Published:** 2019-06

**Authors:** Pratiksha Patel, Priyanka Patel, James E. Meiring, Theresa Misiri, Felistas Mwakiseghile, Melita A. Gordon

**Affiliations:** 1 Malawi Liverpool-Wellcome Trust Clinical Research Programme, Blantyre, Malawi;; 2 Oxford Vaccine Group, Department of Paediatrics, Oxford University, Oxford, United Kingdom;; 3 Institute of Infection and Global Health, University of Liverpool, Liverpool, United Kingdom

The marathon of vaccinating children began on February 21, 2018, at 10:13 am, with 4-year-old Golden becoming the first child in Africa to be enrolled in a typhoid conjugate vaccine study.

The day of Golden’s vaccination was eventful, and all were uttering the same phrase, “27,999 children to be vaccinated by the end of September 2018,” our finish line.

The Typhoid Vaccine Acceleration Consortium (TyVAC) is led by the Center for Vaccine Development and Global Health at the University of Maryland School of Medicine, the Oxford Vaccine Group at the University of Oxford, and PATH. The Typhoid Vaccine Acceleration Consortium is funded by the Bill & Melinda Gates Foundation. This study is part of the TyVAC, a partnership between the Center for Vaccine Development and Global Health at the University of Maryland School of Medicine, the Oxford, and PATH. This vaccine efficacy study is the biggest of its kind to ever be conducted in Blantyre, Malawi.

Our team of doctors, nurses, and field workers were very enthusiastic to meet our goal, an ambitious target of 200 vaccinated children every day. We split ourselves into three vaccination teams. Each team was allocated a school where we vaccinated for a number of days, or even weeks, until all the eligible children were enrolled; then, we would move to a new school. The vaccination areas that we set up varied depending on the space provided by the school. Some provided the entire school hall, some a classroom with desks, whereas others open ground where we would pitch tents. Each time we moved to a new school, we had to set up new vaccination stations in a configuration that ensured smooth flow of children and matched the protocol and standard operating procedures.

The first few weeks we targeted smaller schools, with populations of about 300, where we vaccinated about 100 children per day. Starting in smaller schools helped improve our time management and master the routine on screening, enrolling, and vaccinating the children in the most efficient way possible. We used this experience in larger schools, which had up to 7,000 children.

Parents learned about the project and opportunity to vaccinate their children through letters sent home from school the previous day. We also had a van with a loud speaker on the top that drove around the communities in the evening to spread the news of the study and inform people of the vaccination locations. Parents would bring their school-aged children along with their toddlers and preschool children for vaccination; on average, a mother would bring two to three children, which resulted in good vaccination coverage in all age groups.

We started slow, but soon caught up to a good speed and were vaccinating our target of 200 children per day. However, our recruitment numbers substantially reduced in April when the schools closed down for vacation. We were allowed to continue with vaccination activities despite the holiday, but our means of inviting parents through letters was disrupted. During this period, we spread the news about the study, opportunity to vaccinate, and vaccination locations with our community engagement van. The use of a mobile van was advised by a member of the community advisory group who highlighted that, “the use of a mobile van with key messages would be effective in recruitment for the study. This is still a popular medium by which health, political, and commercial messages are delivered to mass audiences in Blantyre and TyVAC should take advantage of this.”

It was a Monday morning in the second week of April, and like every other day, a few of us reported to our home bases, at Malawi-Liverpool-Wellcome Trust to collect credo boxes containing the vaccine and tablets for recording data, whereas the rest directly reported to the vaccination site and started setting up the stations by arranging furniture stored on site. We drove out through the hills of Ndirande and Zingwangwa through very rocky and bumpy roads to reach the schools, not knowing what effect the van had caused on the turnup of mothers. As we drove onto the school premises, all of us said the same thing, “oh my,” when we saw the huge crowd of mothers. We found the vaccination sessions had already begun. Chrissy, a study nurse, had already given a health talk and explained the study to the mothers. We were all excited and jumped out of the car and took charge of our stations.

Vaccinating children is not as simple as jabbing them one after the other. It involved a lengthy process where mothers who attended the group information session would move with their children through a consent area. Once consented, the child would be assessed for eligibility by the study nurse. Those eligible were given an orange wristband and instructed to queue up at the vaccination station, which was typically a tent or corner of a classroom partitioned using hospital screens to maintain blinding.

The final step was the electronic patient locator station where an electronic mapping tool was used to identify the geographical location, and children were served refreshments. Detailed aerial photographs of Ndirande and Zingwangwa were uploaded onto tablets, and mothers were asked to point to their house on the map. One of us would help using common landmarks such as schools, churches, bars, markets, or mosques. These data enabled estimates of vaccine coverage in the two areas and effective follow-up of participants during the passive surveillance period.

At the end of the day, we packed away all furniture and stored it on site, whereas tablets and vaccines were returned to the Malawi-Liverpool-Wellcome Trust. It was past 7 pm when we got back from the field, and some of us sighed in relief because “it is the end of the day.” For Robert, Cephas, and Innocent, work continued into the evening, as they were the ones driving the van with a loud speaker out to the communities. Returning late required the data team to work late into the night downloading data from all the tablets, reconciling data collected, and charging them so they were ready for the next day.

“You can run a sprint or you can run a marathon but you can’t sprint a marathon” (Ryan Holmes, HootSuite Media, Inc., Vancouver, Canada). The second week of April caused a rush of adrenaline for all of us and was like a 100-m sprint during which we vaccinated 2,000 children in a week with the highest number (636) vaccinated on the 12th of April.

We still had 5 months to complete the study, and the van did wonders with community engagement and recruitment, which brought us back on track. To allow data to be downloaded from the tablets and reconciled and for us to pack up the sites, we returned from the field by 4 pm. This time was sufficient to vaccinate at least 200 children per day. If children turned up late in the afternoon, we advised the mother to come the following morning.

According to Arnold Palmer, “the road to success is always under construction,” and we continued to encounter hurdles further on along the journey in Malawi. We had gone to all schools we were allocated and vaccinated the eligible children before we reached the 28,000 children enrolment number. To address this, we identified new locations situated directly within the communities where the schools were located to use as vaccination sites. Areas such as market places and open grounds close to a chief’s house were selected based on the number of typhoid cases in the previous 3 years. The use of community vaccination sites was an even bigger challenge than vaccinating in schools as we had to use tents that can get very hot because of crowding. I remember a site monitor on a visit commended us for working in such an uncomfortable environment. To ensure blinding was maintained and there was free air circulation, we left one side of the tent open and used hospital screens.

Not only this, but some of the days, we found that the tents had fallen because of heavy rains. We had to pitch them up again, and this delayed our vaccination activities and in turn affected our recruitment numbers.

We were moving closer and closer to September, but the number of children vaccinated per day started to fall again toward the end of August. We were calculating the number of days and number of children remaining to be vaccinated and were unsure if we would reach the target number of enrollees. The recruitment van went out again into the communities to spread the message that it was the last chance for parents to come and enroll their child(ren) in the study. John, the pharmacist wrote a jingle and entertained parents and children as they were waiting. The music and dancing created a fun and positive environment. The van and music were magic. The last mile was a sprint, and in the final 2 weeks of enrolment, more than 300 mothers presented at each vaccination site and more than 100 left without being vaccinated. Mothers would arrive at the vaccination sites as early as 3 and 4 am to ensure they were among the first in the queue. We made it to the finish line on September 27—just 1 day before the intended finish date—having vaccinated a total of 28,143 children and making history in Africa.

